# Patient Selection for Pemafibrate Therapy to Prevent Adverse Cardiovascular Events

**DOI:** 10.3390/jcm12010021

**Published:** 2022-12-20

**Authors:** Toshihide Izumida, Teruhiko Imamura, Nikhil Narang, Koichiro Kinugawa

**Affiliations:** 1 The Second Department of Internal Medicine, University of Toyama, 2630 Sugitani, Toyama 930-0194, Japan; 2 Advocate Christ Medical Center, Oak Lawn, IL 60453, USA

**Keywords:** dyslipidemia, cardiovascular disease, triglyceride

## Abstract

Background: pemafibrate is a newly-introduced selective peroxisome proliferator-activated receptor-α modulator, which decreases serum triglyceride levels with few drug-related adverse events and may reduce the risk of adverse cardiovascular events in carefully selected patients with hypertriglyceridemia. We aimed to understand which specific cohorts may benefit or not from pemafibrate therapy for adverse cardiovascular event risk reduction. Methods: patients with hypertriglyceridemia at baseline received pemafibrate therapy for two years or until October 2022. The factors that were associated with an increased risk of adverse cardiovascular events, defined as heart failure hospitalization, stroke, and acute coronary syndromes, were investigated. Results: a total of 121 patients (median 62 years, 88 men) remained on pemafibrate therapy for a median of 566 days without any drug-related adverse events. During a 3-month therapeutic period, triglyceride levels improved significantly from 302 (205, 581) mg/dL to 178 (117, 253) mg/dL (*p* < 0.001). During the overall therapeutic period, there were nine cardiovascular events. Comorbid chronic heart failure, comorbid coronary disease, and a lower pemafibrate dosing were independently associated with the primary endpoint (*p* < 0.05 for all). Those with multiple risk factors (N = 30) had a significantly higher cumulative incidence of the primary endpoint as compared with others (27% versus 3%, *p* < 0.001). Conclusion: pemafibrate significantly improves hypertriglyceridemia. A higher dose of pemafibrate should be considered to reduce the risk of adverse cardiovascular events, particularly in patients with chronic heart failure or coronary disease.

## 1. Background

Cardiovascular disease, including coronary heart disease, stroke, heart failure, and peripheral artery disease, are leading causes of morbidity and mortality in adults worldwide. Low-density lipoprotein (LDL) cholesterol-lowering therapy using statins reduces the risk of incident cardiovascular disease [1], whereas residual risk remains specifically in patients with hypertriglyceridemia [2]. Fibrates, which activate peroxisome proliferator-activated receptor alpha (PPARα), are used to decrease triglyceride levels, though they have a risk of adverse side effects by way of drug interactions [3]. They can affect hepatic drug-metabolizing enzyme activity and affect the metabolism of statins. Of note, the clinical benefit of these medications, such as fenofibrate and bezafibrate, in decreasing the risk of adverse cardiovascular events has not yet been demonstrated thus far [4]. On the contrary, a sub-group with hypertriglyceridemia, low high density lipoprotein cholesterol, and type 2 diabetes mellitus enjoyed clinical benefit by pemafibrate therapy in the meta-analysis [4].

Pemafibrate is a novel selective PPARα modulator that has recently been introduced with an expected improvement in hypertriglyceridemia and less drug-related adverse events such as drug interaction with statin compared with traditional fenofibrate [5]. The PROMINENT study was a randomized controlled trial to assess the impact of pemafibrate in reducing the risk of adverse cardiovascular events in approximately 10,000 patients with hypertriglyceridemia (serum triglyceride level between 200 and 499 mg/dL), lower high density lipoprotein cholesterol (<40 mg/dL), and diabetes mellitus. The study participants had received moderate- or strong-intensity statin, had low density lipoprotein cholesterol < 70 mg/dL, or had statin intolerance with low density lipoprotein cholesterol < 100 mg/dL. However, the addition of pemafibrate therapy was not superior to the standard of care in this trial [6].

Understanding optimal patient selection for pemafibrate therapy is needed as there may be subgroups that may specifically benefit from this therapy. In this study of patients on pemafibrate therapy, we analyzed baseline patient characteristics that were associated with an increased risk of adverse cardiovascular events.

## 2. Methods

### 2.1. Patient Selection

Consecutive patients who initiated pemafibrate therapy between April 2020 and October 2022 and continued pemafibrate to treat hypertriglyceridemia for 2 years or until October 2022 were retrospectively included.

Patients with serum triglyceride level > 150 mg/dL were considered to receive pemafibrate. Patients with contraindication of pemafibrate, including thyroid function abnormality, severe liver injury, receiving cyclosporine, and maternity, did not receive pemafibrate and were excluded from this study. Patients in whom pemafibrate was initiated as conversion from other conventional fibrates were excluded. Patients who received pemafibrate previously or those who initiated pemafibrate before the study period were excluded. Written informed consents were obtained from all participants before the listing. The institutional review board approved the study protocol (R2015154, 11 April 2016).

### 2.2. Study Protocol

All patients were followed-up from the initiation of pemafibrate (day 0) until the time when pemafibrate was terminated or up to October 2022. The dose of pemafibrate was determined at the discretion of the attending physicians and was fixed in principle during the observational period. The primary endpoint, which was defined beforehand, was the incidence of cardiovascular events defined as heart failure hospitalization, stroke, or acute coronary syndrome during the observational period. We investigated individual baseline patient characteristics that were significantly associated with an increased risk of adverse cardiovascular events.

### 2.3. Baseline Characteristics Data

Baseline characteristics including demographics, comorbidities, and laboratory data were assessed at the time of initiation of pemafibrate (day 0). The laboratory data including lipid parameters were assayed by standard laboratory procedures. All serum and plasma samples were obtained in a fasting condition and frozen at −80 degrees immediately until their assay.

### 2.4. Statistical Analysis

Continuous variables were presented as median (lower quartile, higher quartile) irrespective of their distribution. Categorical variables were presented as numbers and percentages. The continuous variables were compared between the two groups using Mann-Whitney U test. The categorical variables were compared between the two groups using Fischer’s exact test. The association between baseline characteristics and the primary endpoint was investigated by Cox proportional hazard ratio regression analyses. Variables with *p* < 0.05 in the univariable analyses (two-group comparison analyses) were included in the multivariable analysis. Clinically important potential confounders including age, sex, and medications, were also included in the multivariable analysis irrespective of their statistical significance.

A value of *p* < 0.05 was assumed statistically significant. Statistical analyses were performed using SPSS Statistics 23 (SPSS Inc., Armonk, IL, USA).

## 3. Results

### 3.1. Baseline Characteristics

A total of 121 patients were included (Table 1). Median age was 62 (51, 71) years old and 88 (73%) were men. Ten patients (8%) had heart failure and 21 patients (17%) had coronary disease. The baseline triglyceride level was 302 (205, 581) mg/dL. Pemafibrate was initiated at 0.2 mg/day predominantly (94%). Four patients (3%) received 0.1 mg/day and 3 patients (3%) received 0.4 mg/day.

The baseline low-density lipoprotein (LDL) cholesterol level was 109 (66, 124) mg/dL. In this case, 40 patients (33%) received statin and 12 patients (10%) received ezetimibe.

### 3.2. Impact of Pemafibrate on Lipid Parameters

Lipid parameters improved significantly following 3-month pemafibrate therapy (*p* < 0.05 for all), except for statistically unchanged LDL cholesterol levels (*p* = 0.18) (Table 2). Of note, triglyceride levels improved significantly from 302 (205, 581) mg/dL down to 178 (117, 253) mg/dL (*p* < 0.001).

### 3.3. Baseline Characteristics That Were Associated with the Primary Endpoint

During the entire observational period (median 566 [385, 730] days), there were 9 cardiovascular events (4 episodes of coronary revascularization and 5 heart failure hospitalizations). No patients died. All patients continued pemafibrate during the observational period without any drug-related adverse events.

**Figure 1:**
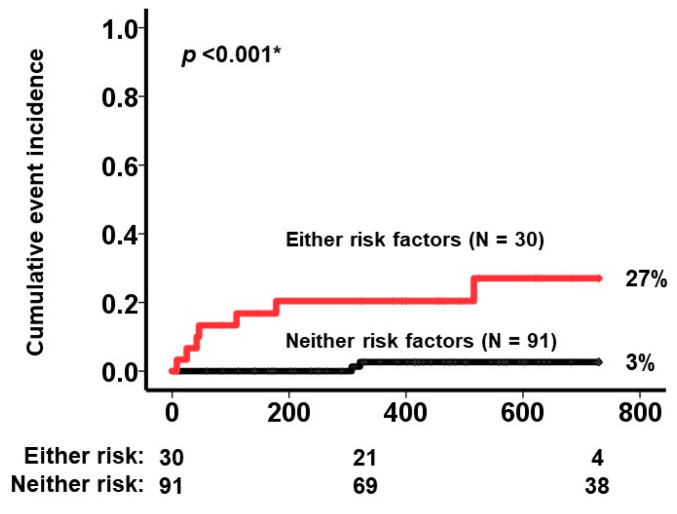
Cumulative incidence of the composite primary endpoint stratified by the existence of three risk factors (either one versus neither one). Risk factors consist of heart failure, coronary disease, and the use of 0.1 mg/day of pemafibrate. Both curves were compared by log-rank test. * *p* < 0.05.

Among baseline characteristics, patients who encountered the events had a higher incidence of chronic heart failure and coronary disease and a lower dose of pemafibrate (*p* < 0.05 for all; Table 1). A history of chronic heart failure, coronary disease, and dose of pemafibrate per 0.1 mg/day decrease were all independently associated with increased risk for the composite primary endpoint (*p* < 0.05 for all; Table 3). In this case, 30 patients satisfied either of the following risk factors: chronic heart failure, coronary disease, and pemafibrate at 0.1 mg/day. They had a significantly higher cumulative incidence of the primary endpoint compared with those without the same baseline characteristics (N = 91) (27% versus 3%, *p* < 0.001; Figure 1). Schoenfeld residuals test confirmed the proportional hazard assumption (*p* > 0.05).

Lipid parameters at 3-month follow-up did not significantly differ between those who experienced the composite endpoint and did not (*p* > 0.05 for all; Table 4). Of note, the triglyceride level was 196 (116, 295) mg/dL in patients with events and 167 (120, 252) mg/dL in patients without events (*p* = 0.60).

## 4. Discussion

We investigated risk factors that were associated with an increased risk of adverse cardiovascular events, defined as heart failure hospitalization, stroke, and acute coronary syndromes, in patients who received pemafibrate therapy to treat hypertriglyceridemia. Overall, pemafibrate therapy improved lipid parameters including serum triglyceride levels. Nevertheless, patients with baseline risk factors including a history of chronic heart failure, coronary disease, and lower dosing of pemafibrate therapy were at increased risk of downstream adverse cardiovascular events.

### 4.1. Improvement in Lipid Parameters by Pemafibrate

As observed in large-scale studies with carefully selected cohorts (randomized control trials and observational studies) or small sample-sized studies [7,8,9], we demonstrated in real-world practice in study cohorts with a multitude of comorbidities that pemafibrate improved lipid parameters, including triglyceride, high-density lipoprotein cholesterol, triglyceride-rich lipoprotein [10], and triglyceride/high-density lipoprotein cholesterol ratio. Of note, the triglyceride/high-density lipoprotein cholesterol ratio is a surrogate of small dense LDL cholesterol [11], which is additionally associated with an increased risk of adverse cardiovascular events independent of LDL cholesterol [12,13].

### 4.2. Factors That Were Associated with Cardiovascular Events

Despite these promising findings, the PROMINENT trial did not demonstrate the benefit of pemafibrate therapy in reducing the risk of adverse cardiovascular events among patients with diabetes mellitus and hypertriglyceridemia [6]. Nevertheless, there may be a subgroup of patients on pemafibrate therapy that would be at high risk of adverse cardiovascular events compared to others due to differences in baseline characteristics. Understanding those with variable risk profiles may help in better designing future trials where this therapy can be studied in targeted cohorts that may experience benefits.

In our study, patients with chronic heart failure and coronary disease were observed to be at higher risk for experiencing adverse cardiovascular events. In the PROMINENT study, a sub-group analysis using the existence of heart failure was not investigated. Similar sub-group analyses were performed in the previous other studies [6]. The FIELD study demonstrated that the history of cardiovascular diseases and age were associated with incremental risk of cardiovascular events during femofibrate therapy [14]. In the ACCORD-lipid study, female sex was associated with incremental risk of coronary events during femofibrate therapy [15].

Despite this, those who experienced events achieved improvement in their hypertriglyceridemia comparably to those who experienced no events. We observed that those on lower doses of pemafibrate were also at increased risk of experiencing adverse cardiovascular events. Median triglyceride level was above 150 mg/dL at follow-up irrespective of the occurrence of adverse events. Although the optimal threshold of serum triglyceride level remains uncertain, aggressive pemafibrate therapy may have a potential role in secondary prevention. Drug-related adverse events at higher dose, particularly renal impairment and thromboembolic events that were higher in the pemafibrate arm in the PROMINENT study, should also be investigated in the next study.

### 4.3. Limitations

Our study included a small cohort size with a short-term observational period. Given the small sample size, we assumed all variables as non-parametric. Given the small event numbers, the numbers of included variables were restricted. We might have missed several potential confounders. This is a proof-of-concept, and further larger-scale studies are warranted to validate and expand our results. We lack a control group and just compared the clinical variables between baseline and 3 months. We used three types of doses (0.1 mg/day, 0.2 mg/day, and 0.4 mg/day) but they were not randomized and just at clinicians’ discretion. We did not assess the impact of pemafibrate on lipid parameters and clinical outcomes. We investigated risk factors for future adverse cardiovascular events during pemafibrate therapy.

## 5. Conclusions

Pemafibrate significantly improves hypertriglyceridemia. Further aggressive pemafibrate therapy might be recommended to reduce the risk of future adverse cardiovascular events, particularly in patients with a history of chronic heart failure and coronary disease.

## Figures and Tables

**Table 1:** The baseline characteristics.

	Total (N = 121)	Events (N = 9)	No Events (N = 112)	*p* Value
Demographics				
Age, years	62 (51, 71)	59 (51, 71)	62 (51, 69)	0.74
Men	88 (73%)	8 (89%)	80 (71%)	0.26
Body mass index	21.2 (20.1, 22.0)	21.1 (20.1, 21.9)	21.3 (20.2, 22.1)	0.33
Comorbidity				
Heart failure	10 (8%)	4 (44%)	6 (5%)	<0.001 *
Atrial fibrillation	13 (11%)	1 (11%)	12 (11%)	0.97
Diabetes mellitus	70 (58%)	3 (33%)	67 (60%)	0.12
Coronary disease	21 (17%)	4 (44%)	17 (15%)	0.026 *
History of stroke	14 (12%)	0 (0%)	14 (13%)	0.26
Dose of pemafibrate				<0.001 *
0.1 mg/day	4 (3%)	3 (33%)	1 (1%)	-
0.2 mg/day	114 (94%)	6 (67%)	108 (96%)	-
0.4 mg/day	3 (3%)	0 (0%)	3 (3%)	-
Concomitant medication				
Statin	40 (33%)	4 (44%)	36 (32%)	0.45
Ezetimib	12 (10%)	1 (11%)	11 (10%)	0.90
Anti-platelet	29 (24%)	3 (33%)	26 (23%)	0.49
Renin-angiotensin-aldosterone inhibitor	72 (60%)	5 (56%)	67 (60%)	0.80
Laboratory data				
Hemoglobin, g/dL	14.3 (12.3, 15.7)	13.3 (11.8, 15.5)	14.5 (12.7, 15.9)	0.52
Estimated GFR, mL/min/1.73m^2^	63.4 (39.3, 75.3)	62.5 (56.4, 67.1)	63.4 (38.9, 77.0)	0.084
Total cholesterol, mg/dL	201 (165, 227)	182 (137, 206)	204 (167, 228)	0.21
LDL-cholesterol, mg/dL	109 (66, 124)	71 (53, 124)	111 (74, 126)	0.13
HDL-cholesterol, mg/dL	44 (35, 46)	38 (36, 41)	44 (34, 47)	0.084
Triglyceride, mg/dL	302 (205, 581)	428 (201, 651)	302 (207, 466)	0.62
Triglyceride/HDL-cholesterol ratio	7.0 (4.6, 16.1)	11.4 (4.7, 17.0)	7.0 (4.5, 14.5)	0.37
Triglyceride-rich lipoprotein, mg/dL	49 (33, 69)	53 (33, 70)	49 (33, 68)	0.63

Continuous variables are stated as median and interquartile. Categorical variables are stated as numbers and percentage. Continuous variables are compared between the two groups using Mann-Whitney U test. Categorical variables are compared between the two groups using Fischer’s exact test. GFR, glomerular filtration ratio; LDL, low-dense lipoprotein; HDL, high-dense lipoprotein. * *p* < 0.05.

**Table 2:** The trends in lipid parameters during 3-month pemafibrate therapy.

	Baseline	3 Months Follow-Up	*p* Value
Total cholesterol, mg/dL	201 (165, 227)	182 (158, 212)	<0.001 *
LDL-cholesterol, mg/dL	109 (66, 124)	104 (88, 136)	0.18
HDL-cholesterol, mg/dL	44 (35, 46)	49 (37, 52)	<0.001 *
Triglyceride, mg/dL	302 (205, 581)	178 (117, 253)	<0.001 *
Triglyceride/HDL-cholesterol ratio	7.0 (4.6, 16.1)	4.3 (2.5, 5.7)	<0.001 *
Triglyceride-rich lipoprotein, mg/dL	49 (33, 69)	27 (22, 45)	<0.001 *

Variables are stated as median and interquartile and compared between the two timings using Wilcoxon signed-rank test. LDL, low-dense lipoprotein; HDL, high-dense lipoprotein. * *p* < 0.05.

**Table 3:** The predictors for the composite primary endpoint among baseline characteristics.

Variables	Hazard Ratio (95% Confidence Interval)	*p* Value
Heart failure	7.38 (1.72–35.3)	0.012 *
Coronary disease	7.76 (1.69–34.3)	0.013 *
Dose of pemafibrate as 0.1 mg/day decrease	17.6 (3.13–94.4)	0.001 *
Age, years	1.02 (0.96–1.07)	0.58
Male sex	3.21 (0.40–25.6)	0.27
Anti-platelet	0.96 (0.94–10.5)	0.44
Renin-angiotensin-aldosterone inhibitor	0.97 (0.95–13.1)	0.27

* *p* < 0.05 by Cox proportional hazard ratio regression analyses.

**Table 4:** The lipid parameters at 3-month follow-up stratified by experiencing an adverse cardiovascular event.

	Events (N = 9)	No Events (N = 112)	*p* Value
Total cholesterol, mg/dL	171 (151, 201)	188 (162, 218)	0.37
LDL-cholesterol, mg/dL	98 (79, 116)	106 (83, 134)	0.51
HDL-cholesterol, mg/dL	42 (37, 49)	49 (40, 55)	0.34
Triglyceride, mg/dL	196 (116, 295)	167 (120, 252)	0.60
Triglyceride/HDL-cholesterol ratio	5.6 (3.1, 6.0)	3.4 (2.4, 5.6)	0.36
Triglyceride-rich lipoprotein, mg/dL	36 (27, 42)	30 (24, 46)	0.48

Variables are stated as continuous variables and are compared between the two groups using Mann-Whitney U test. LDL, low-dense lipoprotein; HDL, high-dense lipoprotein.

## Data Availability

Data including study protocol are available from the corresponding authors upon reasonable request.
